# Identification and validation of a novel angiogenesis-related gene signature for predicting prognosis in gastric adenocarcinoma

**DOI:** 10.3389/fonc.2022.965102

**Published:** 2023-01-16

**Authors:** Peipei Xu, Sailiang Liu, Shu Song, Xiang yao, Xuechuan Li, Jie Zhang, Yinbing Liu, Ye Zheng, Ganglong Gao, Jingjing Xu

**Affiliations:** ^1^ Department of Biliary-Pancreatic Surgery, Renji Hospital Affiliated to Shanghai Jiao Tong University, School of Medicine, Shanghai, China; ^2^ State Key Laboratory of Oncogenes and Related Genes, Shanghai, China; ^3^ Department of Pathology, Shanghai Public Health Clinical Center Affiliated to Fudan University, Shanghai, China

**Keywords:** angiogenesis-related gene, long ncRNAs, gastric adenocarcinoma, prognosis, immunotherapy

## Abstract

**Background:**

Angiogenesis is a major promotor of tumor progression and metastasis in gastric adenocarcinoma (STAD). We aimed to develop a novel lncRNA gene signature by identifying angiogenesis-related genes to better predict prognosis in STAD patients.

**Methods:**

The expression profiles of angiogenesis-related mRNA and lncRNA genes were collected from The Cancer Genome Atlas (TCGA). Then, the “limma” package was used to identify differentially expressed genes (DEGs). The expression profiles of angiogenesis-related genes were clustered by consumusclusterplus. The Pearson correlation coefficient was further used to identify lncRNAs coexpressed with angiogenesis-related clustere genes. We used Lasso Cox regression analysis to construct the angiogenesis-related lncRNAs signature. Furthermore, the diagnostic accuracy of the prognostic risk signature were validated by the TCGA training set, internal test sets and external test set. We used multifactor Cox analysis to determine that the risk score is an independent prognostic factor different from clinical characteristics. Nomogram has been used to quantitatively determine personal risk in a clinical environment. The ssGSEA method or GSE176307 data were used to evaluate the infiltration state of immune cells or predictive ability for the benefit of immunotherapy by angiogenesis-related lncRNAs signature. Finally, the expression and function of these signature genes were explored by RT–PCR and colony formation assays.

**Results:**

Among angiogenesis-related genes clusters, the stable number of clusters was 2. A total of 289 DEGs were identified and 116 lncRNAs were screened to have a significant coexpression relationship with angiogenic DEGs (P value<0.001 and |R| >0.5). A six-gene signature comprising LINC01579, LINC01094, RP11.497E19.1, AC093850.2, RP11.613D13.8, and RP11.384P7.7 was constructed by Lasso Cox regression analysis. The multifactor Cox analysis and Nomogram results showed that our angiogenesis-related lncRNAs signature has good predictive ability for some different clinical factors. For immune, angiogenesis-related lncRNAs signature had the ability to efficiently predict infiltration state of 23 immune cells and immunotherapy. The qPCR analysis showed that the expression levels of the six lncRNA signature genes were all higher in gastric adenocarcinoma tissues than in adjacent tissues. The functional experiment results indicated that downregulation of the expression of these six lncRNA signature genes suppressed the proliferation of ASG and MKN45 cells.

**Conclusion:**

Six angiogenesis-related genes were identified and integrated into a novel risk signature that can effectively assess prognosis and provide potential therapeutic targets for STAD patients.

## Introduction

Gastric cancer (GC) is the fifth most frequent type of cancer and the fourth leading cause of cancer-related deaths worldwide (1). Stomach adenocarcinoma (STAD) is the most common histological type and accounts for 95% of GC (2). In 2020, GC was expected to cause over one million new cases and an estimated 769,000 deaths. Despite decades of advances in diagnostic and treatment techniques, STAD mortality remains high, and 5-year survival remains unsatisfactory. This is urgently needed to identify new and effective potential diagnostic biomarkers and develop new therapeutic approaches.

Angiogenesis is a multistep process triggered by multiple biological signals (3). Angiogenesis can be regulated by the balance of growth and inhibitory factors in healthy tissue. As this balance is disrupted, too much or too little angiogenesis occurs, leading to various diseases, including malignant tumors (4). Tumors need to build new blood vessels to continue growing and thus need to be triggered by chemical signals from tumor cells that stimulate angiogenesis, thereby promoting tumor growth (5). In the absence of vascular support, tumors may undergo necrosis or even apoptosis (6).

Long ncRNAs (lncRNAs), with a length of more than 200 nucleotides, are defined as a large and heterogeneous class of regulatory transcripts and are transcribed from the genome but generally lack protein-coding potential (7). Accumulating evidence suggests that lncRNAs are not only key regulators of cancer pathways but also biomarkers of disease (8, 9). LncRNAs have been shown to perform multiple functions associated with the classic hallmarks of cancer, including accelerated proliferation, immune escape, induction of angiogenesis, and drug resistance (10). Moreover, increasing evidence has revealed that abnormally expressed lncRNAs are found in STAD (11). In addition to their possible roles in cancer biology, lncRNAs have emerged as a new class of promising diagnostic and prognostic biomarkers. Compared with mRNA, the expression of lncRNAs is more tissue specific, which may provide new information for finding specific biomarkers for STAD (12). For example, the 5-lncRNA model can serve as an accurate signature to predict the prognosis of patients with liver cancer (13). The 7 lncRNAs (AC017048.3, ADAMTSL4‐AS1, AL035209.2, RP11‐16M8.2, RP11‐384O8.1, RP11‐462G12.1, and RP11‐476D10.1) were identified to predict lung adenocarcinoma patient survival as independent prognostic biomarkers (14). Li X et al. identified 5 survival-related lncRNAs and validate their clinical significance, angiogenesis correlation and prognosis-predictive values, which may provide a new perspective and some promising antiangiogenic targets for clinical diagnosis and treatment strategies of bladder urothelial carcinoma (15). However, studies on the signature of angiogenesis-related lncRNAs during STAD survival are still lacking.

In the present study, we discovered and validated an angiogenesis-related lncRNA signature to predict prognosis in patients with STAD. Additionally, we constructed an angiogenesis-related lncRNA risk scoring model based on six lncRNA characteristics and clinical factors. Finally, we further evaluated the effect of the risk scoring model on clinical characteristics, tumor mutational burden, immune cell infiltration, and predictive power in the context of immunotherapy benefit. In summary, we established a prognostic signature consisting of six angiogenesis-related genes for STAD patients, which was verified as a key prognostic predictor and may serve as a potential therapeutic target for STAD.

## Materials and methods

### Data set source and preprocessing

Gene expression data and complete clinical annotations were obtained from The Cancer Genome Atlas Database (TCGA). Gene expression RNA sequencing data (FPKM value) and clinical information were downloaded from UCSC Xena (https://gdc.xenahubs.net). Based on clinical information, samples with missing overall survival time or 0 days were excluded. The preprocessed TCGA_STAD data set has a total of 350 tumor samples. The list of angiogenesis-related genes was obtained from hallmark gene sets in the molecular signature database. A total of 36 genes were included in the analysis.

### Unsupervised clustering of angiogenesis-related genes

The “Consensus Cluster Plus” software package (16) (http://www.bioconductor.org/) was used to clarify the biological characteristics of angiogenesis-related genes and treat STAD patients divided into different subtypes. Principal component analysis was used to evaluate gene expression patterns between different STAD subtypes. The Kaplan–Meier method was used to detect survival curves and compare survival differences among different subgroups.

### Differential expression analysis between different subtypes

According to the results of consistent clustering, the tumor samples were divided into two groups, cluster A and cluster B. The limma package of R software was used to analyse the differential expression of genes between the angiogenic subtypes of TCGA_STAD tumor samples (17). The screening threshold for differential gene expression was set to FDR<0.05 and | log2(FC)|>1. The annotation file (*. GTF) of Ensemble was used to extract lncRNAs (18).

### Construction of a lncRNA risk scoring model related to angiogenesis

In this study, we analysed the correlation between lncRNAs and angiogenesis-related genes in the TCGA_STAD data set. A tumor risk score model was constructed based on angiogenesis-related lncRNAs. First, to reduce noise or redundant genes, the single-factor Cox algorithm was used to reduce the size of the lncRNA gene set. After the reduction, the Lasso (least absolute shrinkage and selection operator, Tibshirani (1996)) method is used to filter the variables to reduce the number of genes in the risk model (19). Finally, a multifactor Cox regression model was used to construct a risk score model for tumor immune cell infiltration. The calculation formula is as follows:


Risk_scores=∑Coef(i)*Exp(i)

### Estimation of tumor immune microenvironment (TIME) cell infiltration

We used the single sample gene set enrichment analysis (ssGSEA) algorithm to quantify the relative abundance of infiltrating cells in STAD_TIME (20). We obtained a set of genes that mark each TIME-infiltrating immune cell type, which is rich in a variety of human immune cell subtypes, including activated CD8 T cells, activated dendritic cells, macrophages, natural killer T cells, and regulatory T cells. The enrichment score calculated by ssGSEA was used to represent the relative abundance of each TIME infiltrating cnnell in each sample.

### Genome Variation Analysis (GSVA)

To study the differences in biological processes between different angiogenesis subtypes, we used the “GSVA” R package to perform GSVA enrichment analysis (21). GSVA is a nonparametric and unsupervised method usually used to estimate pathway and biological process activity changes in expression data set samples. “C2.cp.kegg.v7.2. “Symbols” were downloaded from the MSigDB database and used to perform GSVA (22). The R package “limma” was used to calculate differential expression pathways. FDR<0.05 and |log2FC|>0.2 were set as the cut-offs.

### Tissue specimens

Fresh STAD tissues and adjacent normal tissues were collected from Renji Hospital, Shanghai Jiaotong University School of Medicine. No patients received treatment before surgery, and all patients signed informed consent forms provided by Renji Hospital, Shanghai Jiaotong University School of Medicine. The primary tumor and normal surgical marginal tissue were immediately isolated from each patient by an experienced pathologist and stored in liquid nitrogen until use. The study was approved by the Ethics Committee of Renji Hospital (RA-2021-024), Shanghai Jiaotong University School of Medicine.

### Cell culture, transfection and transduction

The human gastric cancer cell lines AGS and MKN45 were obtained from Shanghai Institute of Digestive Disease, Renji Hospital, School of Medicine, Shanghai Jiao Tong University. The cell lines were cultured in DMEM supplemented with 10% fetal bovine serum (Invitrogen, San Diego, CA) at 37°C under 5% CO2 in a humidified incubator. Human-specific siRNA sequences are shown in **Supplementary Table S1**. The oligonucleotides were chemically synthesized by GenePharma (Shanghai, China). The transfection method was described in our previous article.

### Total RNA extraction and quantitative real−time PCR

RNA extraction from cells was performed with TRIzol reagent according to a standard protocol. A total of 1 μg DNase-treated RNA was reverse-transcribed into cDNA using the QuantiTect Reverse Transcription Kit (QIAGEN, Valencia, CA, USA). Two negative controls were set in the experiment, including one without template RNA and the other without reverse transcriptase. The 6-gene signature comprising LINC01579, LINC01094, RP11.497E19.1, AC093850.2, RP11.613D13.8, and RP11.384P7.7 was also quantified by SYBR-Green q-PCR and normalized to the levels of GAPDH. The sequences of upstream and downstream primers are shown in **Supplementary Table S2**. All PCRs were performed in triplicate. All independent experiments were performed in triplicate.

### Clone information

The soft agar colony formation assay was applied to evaluate the ability of a single cell to grow into a colony. Cells were seeded into 6-well plates at 300 cells per plate. The cells were mixed and then cultured for 7-10 days in culture media with 10% FBS. Cell clusters of more than 30 cells were counted as colonies. All independent experiments were performed in triplicate.

### Statistical analysis

We used R version 3.5.3 for statistical analysis. The Wilcoxon test was used to compare the expression levels of angiogenic factors between TP53 and TTN mutant samples and wild-type samples. The survival curve was generated by the Kaplan–Meier method. The difference between groups was compared by the log rank test. A Cox regression model was used for single-factor and multivariate analyses to determine the independent prognostic value of the risk score. We used the ROC curve to estimate the predictive efficiency of the risk model for 1-year, 3-year, and 5-year OS. P<0.05 was considered statistically significant. Where applicable, data are presented as the mean ± SEM from at least three replicates. All independent experiments were performed in triplicate. Differences were considered statistically significant when P values were less than 0.05. All data were analysed using SPSS 13.0 statistical software (SPSS Inc., Chicago, IL, USA).

## Results

### Molecular characteristics of angiogenesis genes in gastric adenocarcinoma

To clearly illustrate the process of our research, a flow chart is shown in **Supplementary Figure S1**.

**Figure 1:**
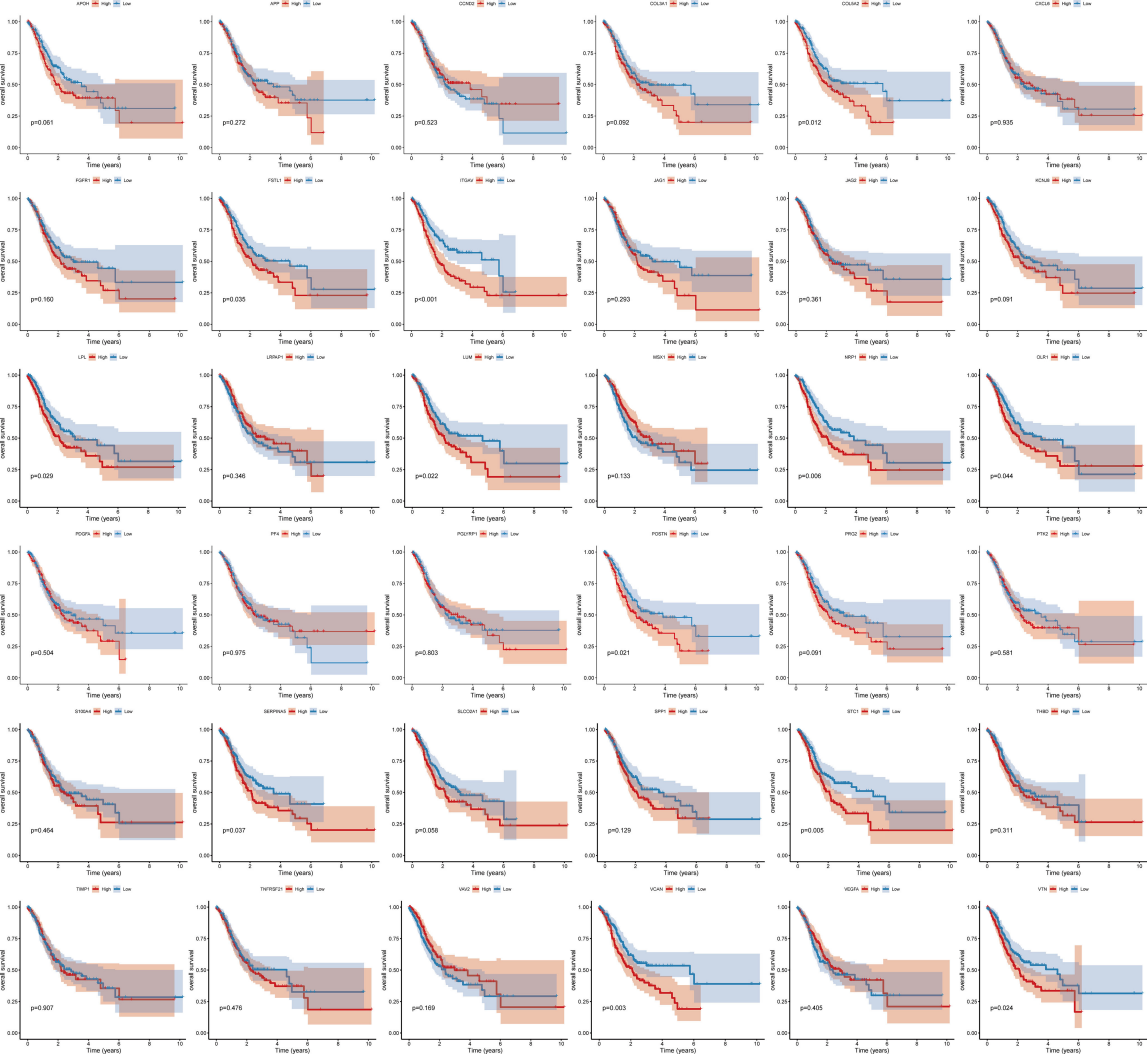
Survival curve of angiogenesis-related genes.

**Figure 2:**
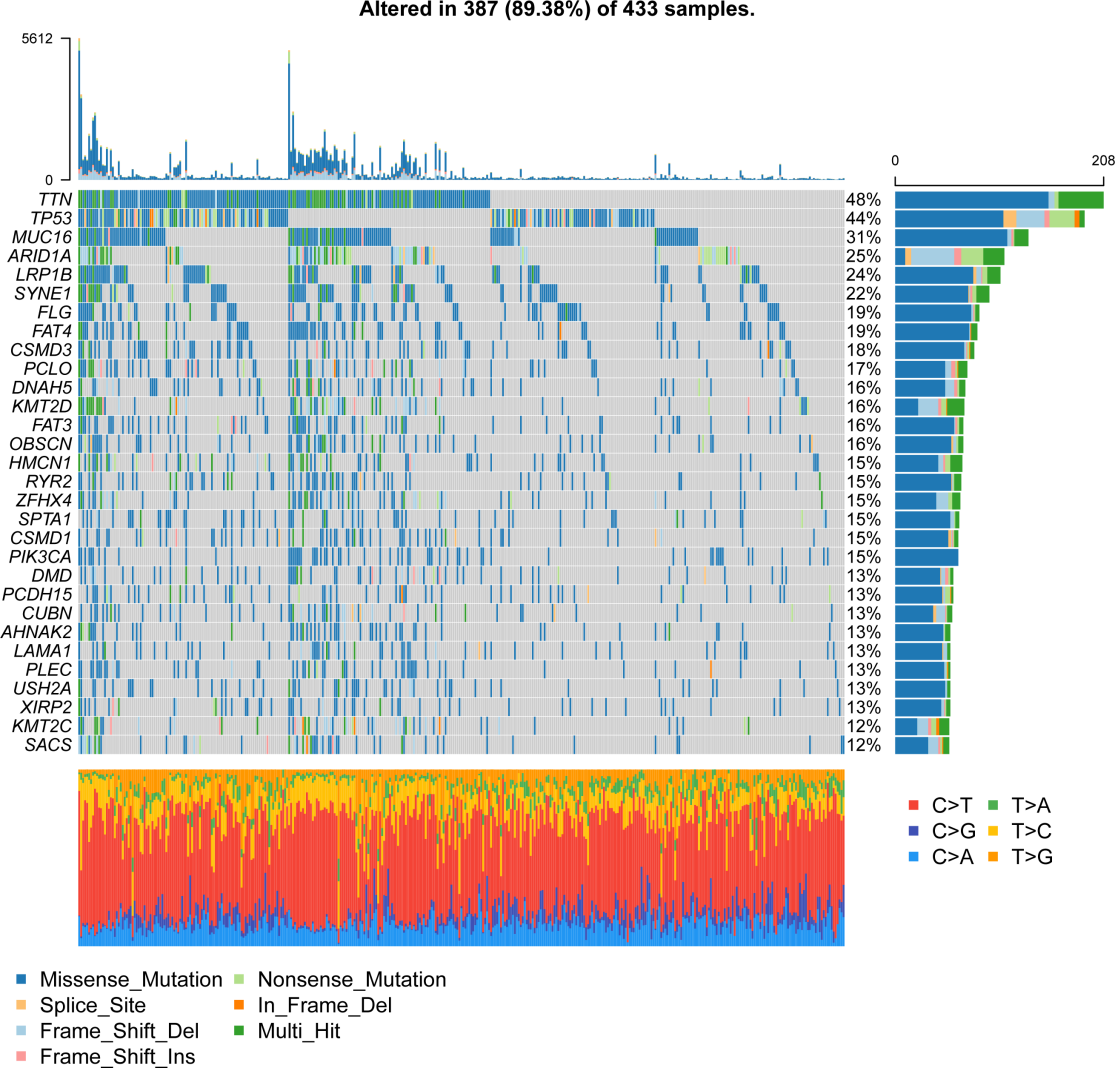
Waterfall plot of gene mutations in the TCGA_STAD dataset.

To study the molecular characteristics of angiogenesis genes of GC, 36 angiogenesis-related genes were divided into high expression and low expression by the median expression of the TCGA_STAD data set. First, KM Curve were used to screen differentially expressed genes meaningful for survival. The results showed that low expression of COL5A2, FSTL1, ITGAV, LPL, LUM, NRP1, OLR1, POSTN, SERPINA5, STC1, VCAN and VTN was associated with a better OS prognosis (**Figure 1**). Subsequently, we analysed the mutations of 36 angiogenesis-related genes, and found that the overall mutation rate of angiogenesis-related genes varied to varying degrees in the genome. The statistics of gene mutations in the TCGA_STAD data set showed that 89.38% of tumor samples had gene mutations. Among them, the TTN, TP53, MUC16 and ARID1A genes had relatively high mutation ratios of 48%, 44%, 40%, 31% and 25%, respectively (**Figure 2**).

**Figure 3:**
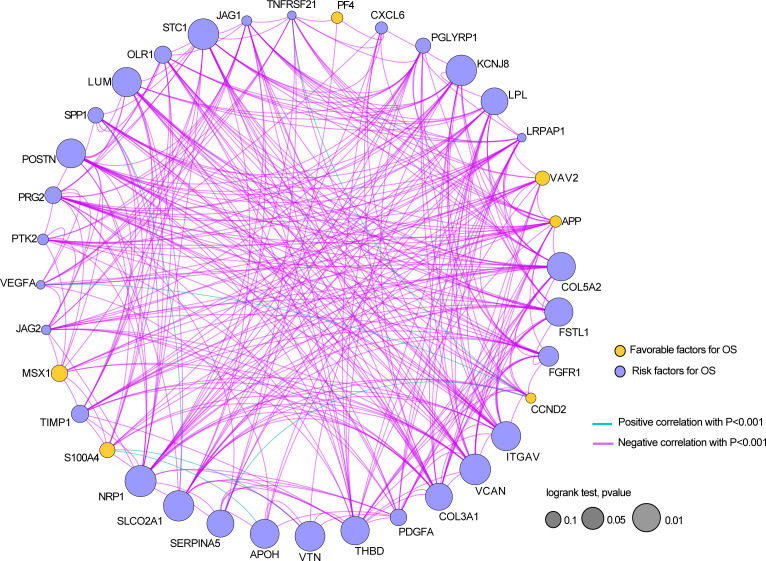
The relationship between the expressions of 36 angiogenesis-related genes in the STAD dataset.

Next, we analyzed the coexpression of 36 angiogenesis-related genes and found that there was a significant negative correlation between most genes, as shown in **Figure 3**. Furthermore, a hypothesis test was conducted to determine whether TP53 and TTN affect the expression of 36 angiogenesis-related genes. The results showed that mutation of the TTN gene was significantly correlated with high expression of the LRPAP1, PTK2, and VAV2 genes and correlated with low expression of the CCND2, FSTL1, KCNJ8, LPL, LUM, NRP1, SERPINA5, SLCO2A1, STC1, THBD and VTN genes (**Supplementary Figure S2**). Among the mutation groups with the TP53 gene, APOH, JAG2, PTK2, VAV2, and VEGFA genes have a significantly high expression state, while APP, CCND2, COL3A1, COL5A2, FGFR1, FSTL1, ITGAV, KCNJ8, LRPAP1, LUM, NRP1, OLR1, S100A4, TIMP1, and VCAN genes showed a significantly low expression state (**Supplementary Figure S3**).

In conclusion, the above results revealed that these angiogenesis-related might play crucial roles in GC.

### Identification of angiogenic subtypes and differentially expressed genes in gastric adenocarcinoma

**Figure 4:**
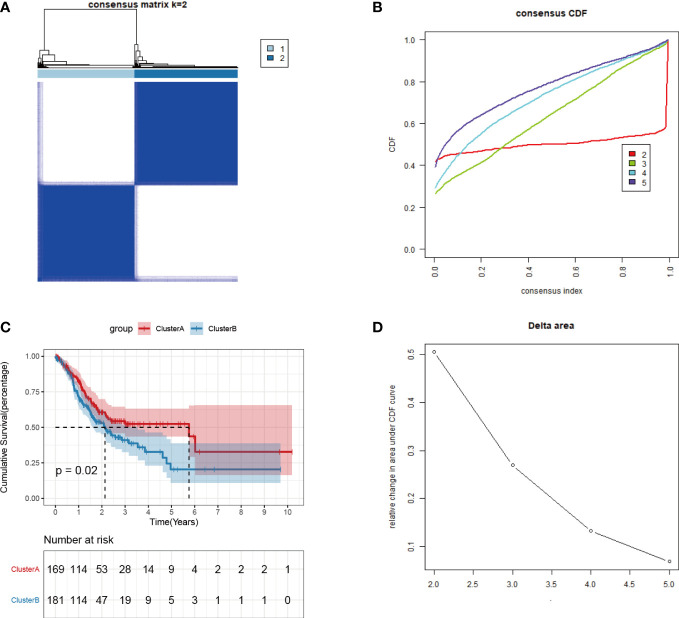
Consistent clustering of tumor angiogenesis-related gene expression profiles. **(A)**Distribution of consensus cumulative distribution function; **(B)** Cumulative distribution function delta curve; **(C)** Distribution of consensus matrix k=2; **(D)** Prognostic features of two DNA methylation subtypes.

**Figure 5:**
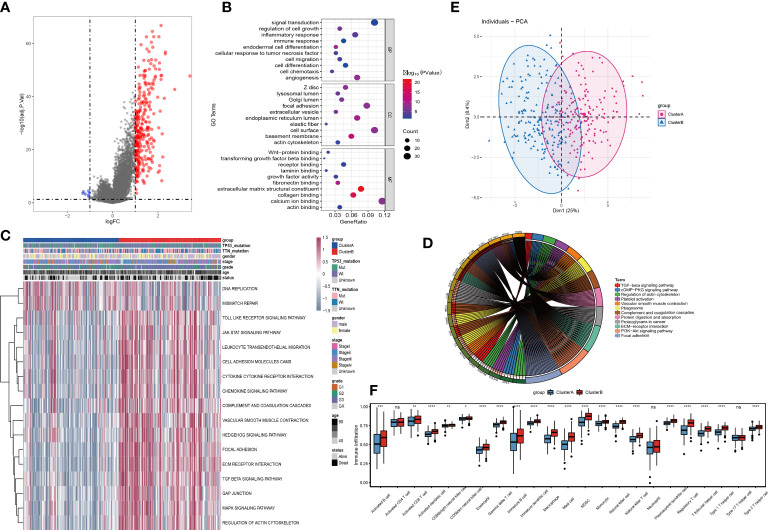
Identification and functional analysis of differentially expressed genes among tumor angiogenesis subtypes. **(A)**Volcano plot for differential expression; **(B)**Bubble plot for GO enrichment analysis of differentially expressed genes; **(C)**Differential expression pathways for GSVA analysis; **(D)** PCA analysis for expression profiles; **(E)** KEGG enrichment analysis for differentially expressed genes Circle; **(F)** Analysis of tumor immune cell infiltration. Differences between the two groups were analyzed using the independent t-test, values are expressed as the means±sem. ns, P>0.05; *P < 0.05; **P < 0.01; ***P < 0.001; ****P < 0.0001.

The expression profiles of 36 angiogenesis-related genes were clustered by consensus clustering (consumusclusterplus). The optimal number of clusters was determined according to the cumulative distribution function (CDF), and the CDF delta area curve was observed in k=2 (**Figure 4A**). Further analysis of the prognostic features of these angiogenesis-related genes clusters revealed significant prognostic differences in k=2 (**Figure 4B**). Among these angiogenesis-related genes clusters, relatively stable clustering results were observed using a cluster of 2 (**Figure 4C**). The analysis of the prognostic features revealed that angiogenesis-related genes cluster B was associated with a poor prognosis (**Figure 4D**). To reveal the potential biological characteristics of different angiogenesis subtypes, the limma package of R software was used to analyse the differential expression of genes in the 2 angiogenesis-related genes clusters in TCGA-STAD. The screening threshold was set to p<0.05 and | log2(Fold Change) | >1. A total of 289 differentially expressed genes were identified, of which 282 genes were highly expressed in cluster B and 7 genes were highly expressed in cluster A (**Figure 5A**). Subsequently, functional enrichment analysis of GO was performed on the differentially expressed genes between the 2 clusters (**Figure 5B**). The first 10 pathways enriched in the three functional classifications (BP, CC, MF) are displayed with a bubble chart, and the results showed that differentially expressed genes were mainly distributed on the cell surface, in focal adhesions, and in the endoplasmic reticulum cavity. To further explore the relationship between angiogenesis-related genes clusters, and the principal component analysis (PCA) algorithm was used to visualize the expression profile related to angiogenesis-related genes. The samples had a good aggregation form in the space of the first and second dimensions (**Figure 5C**).

Further, the GSVA results showed that the 2 clusters have significant differences in signal pathways such as DNA replication, mismatch repair, JAK STAT, cytokine receptor interaction, chemokines, TGF BETA, and MAPK pathways (**Figure 5D**). We also performed KEGG enrichment analysis and found that the differential genes still play a role in TGF beta pathway, regulation of actin cytoskeleton, vascular smooth muscle contract and other signal pathways (**Figure 5E**). Then, by analysing the expression differences of various immune-infiltration factors between cluster A and cluster B (**Figure 5F**), it was found that there were significant differences in most categories. Among them, Activated B cell, Activated CD8 T cell, Activated dendritic cell, CD56 bright natural killer cell, CD56 dim natural killer cell, Eosinophil, Gamma delta T cell, Immature B cell, Immature dendritic cell, Macrophage, Mast cell, MDSC, Monocyte, Natural killer cell, Natural killer T cell, Neutrophil, Plasmacytoid dendritic cell, Regulatory T cell, T follicular helper cell, Type 1 T helper cell and Type 2 T helper cell have significant differences between cluster A and cluster B, while Activated CD4 T cell and Type 17 T helper cell have no differences between cluster A and cluster B.

### Construction of lncRNA models related to angiogenesis in gastric adenocarcinoma

**Figure 6:**
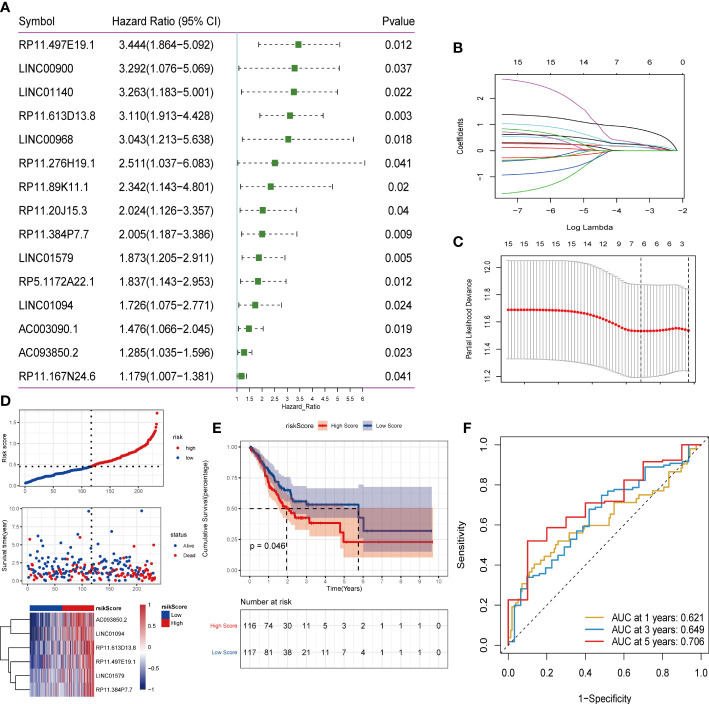
Screening of lncRNA and construction of risk model. **(A)** One-factor cox screening; **(B)** Change trajectory of each independent variable, the horizontal axis represents the log value of the independent variable lambda, and the vertical axis represents the coefficient of the independent variable; **(C)** Confidence interval under each lambda; **(D)** Risk Distribution of scores; **(E)** Survival curve; **(F)**ROC curve.

To explore the expression of angiogenesis-related lncRNA genes and their role in the prediction of overall tumor survival, the Pearson correlation coefficient was further used to identify lncRNAs coexpressed with angiogenesis-related genes (P value<0.001 and |R| >0.5). A total of 116 lncRNAs were screened to have a significant coexpression relationship with at least one angiogenic gene. In this study, the risk score model of STAD was constructed based on the coexpressed lncRNAs of angiogenesis-related genes. First, the overall set of TCGA_STAD (n=350) was divided into a training set (n=233) and a test set (n=117) according to an approximate 2:1 ratio. In the training set, 116 candidate lncRNAs were identified by single-factor Cox analysis. The threshold was set to p value<0.05, and 15 lncRNAs were retained (Table S10, **Figure 6A**). Then, Lasso regression was used to solve the multicollinearity problem during regression analysis and screen the variables in the risk model. We used the glmnet package to perform Lasso Cox regression analysis and the change trajectory of each independent variable (**Figure 6B**). As lambda gradually increases, the number of independent variable coefficients tends to gradually increase. Next, we used a 10-fold cross test to construct the model and confidence interval under each lambda (**Figure 6C**).

The final 6-lncRNA gene signature formula is as follows: RiskScore = (0.153)* AC093850.2 + (0.159)* LINC01094 + (0.245)* LINC01579 + (0.268)* RP11.384P7.7 + (0.327)* RP11.497E19.1 + (0.878)* RP11.613D13. 8. Otherwise, we conducted survival analysis on these six genes based on TCGA database, shown in (**Supplementary Figure S4**). Kaplan–Meier analysis showed that these six genes in the high-level group had a worse prognosis than in the low-level groups.

Furthermore, we judged the impact of risk scores constructed by 6 lncRNAs on overall survival. First, according to the median risk score, the samples were divided into high-risk groups and low-risk groups. It can be observed that the high-risk group had a higher proportion of death samples (**Figure 6D**). Kaplan–Meier analysis of the risk score between the high- and low-risk groups showed that the overall survival (OS) of patients in the high-risk score group was significantly lower than that of patients in the low-risk score group (**Figure 6E**). Risk scores have a good ability to predict the overall survival of the TCGA_STAD data set. To investigate the diagnostic accuracy of the prognostic risk model, the areas under the time-dependent ROC curves (AUCs) were computed. The 1-, 3-, and 5-year AUCs were 0.621, 0.649, and 0.706, respectively (**Figure 6F**).

**Figure 7:**
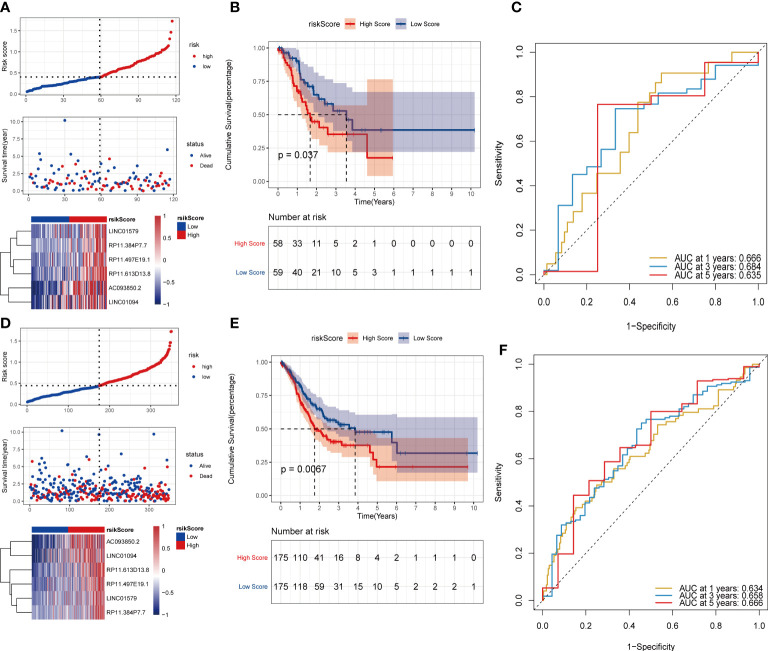
Validation of the risk model on the test set and the ensemble set. **(A)**Distribution map of test set risk scores; **(B)** Test set survival curve; **(C)** Test set 1, 3, and 5-year ROC curves; **(D)** Distribution map of overall set risk score; **(E)** Overall set survival curve; **(F)** Overall set 1, 3, 5-year ROC curve.

Subsequently, the test set and the overall set of TCGA_STAD were used to test the predictive ability of risk scores on OS. First, based on the same algorithm, the risk scores of each sample are calculated in the test set and the overall set. Subsequently, according to the median risk scores, the samples were divided into high-risk groups and low-risk groups. It can also be observed that the high-risk group had a higher proportion of death samples in the test set (**Figure 7A**) and the overall set (**Figure 7D**). Kaplan–Meier analysis of the risk score between the high- and low-risk groups showed that the overall survival of patients in the high-risk-score group was significantly lower than that of patients in the low-risk-score group, as shown in the test set (**Figure 7B**) and the overall set (**Figure 7E**). In the TCGA_STAD test set, the risk score value had a good ability to predict the overall survival of the test set, and the AUCs at 1, 3, and 5 years were 0.666, 0.684, and 0.635, respectively (**Figure 7C**). Similarly, in the overall data set of TCGA_STAD, the risk score value also had a good ability to predict the overall survival of the overall set, and its 1-, 3-, and 5-year AUCs were 0.634, 0.658, and 0.666, respectively (**Figure 7F**).

**Figure 8:**
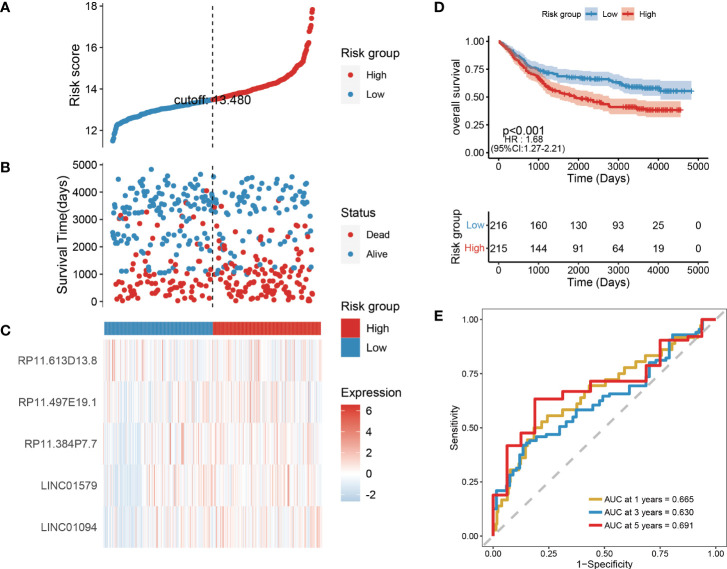
Validation of risk models on external datasets. **(A–C)** Distribution map of risk scores in colon cancer; **(D)** Survival curve in colon cancer; **(E)** 1, 3, and 5-year ROC curve in colon cancer.

To further evaluate the robustness of the risk scores constructed by 6 lncRNAs in predicting the OS of tumors, this study selected the colon cancer (TCGA_COAD) data set, which is also a gastrointestinal tumor, in the TCGA database for analysis. Using the same formula, we calculated the risk score of the tumor samples in TCGA_COAD and divided the samples into high-risk groups and low-risk groups according to the median risk score. High-risk groups were also observed in TCGA_COAD, and the proportion of death samples was relatively high (**Figures 8A–C**). Kaplan–Meier analysis of the risk score between the high- and low-risk groups showed that the overall survival of patients in the high-risk-score group was significantly lower than that of patients in the low-risk-score group (**Figure 8D**). In the TCGA_COAD data set, the risk score value has a good ability to predict the overall survival time. Its 1-, 3-, and 5-year AUCs were 0.665, 0.630, and 0.691, respectively (**Figure 8E**).

### The relationship between risk assessment and clinical characteristics

**Figure 9:**
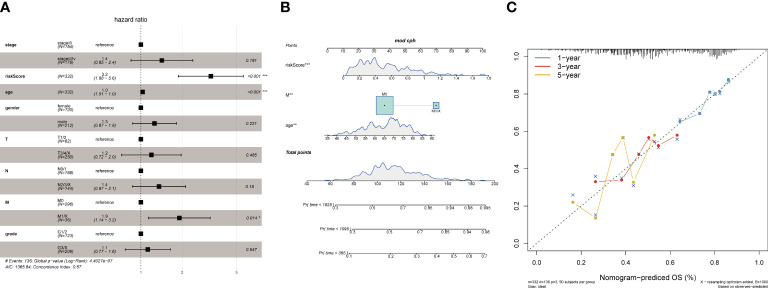
Association of tumor risk scores with clinical characteristics. **(A)**Multivariate Cox analysis of clinical features and risk scores; **(B)** Nomograms of clinical features and risk scores; **(C)** Nomograms for 1, 3, and 5 years.

Age and tumor grade are important clinical information for sample patients. It is necessary to clarify the relationship between the tumor risk score and clinical characteristics. First, we used multifactor Cox analysis to determine that the risk score is an independent prognostic factor different from age, sex, smoking status, stage, M stage, N stage, and T stage. The results indicated that the risk score was an independent prognostic factor for OS (p< 0.05) in the TCGA-STAD database (**Figure 9A**). According to the results of multivariate analyses, we constructed a nomogram model with clinical features, which is a powerful tool that has been used to quantitatively determine personal risk in a clinical environment. We constructed a nomogram by combining risk score, age, and M stage to predict the probability of 1-year, 3-year, and 5-year OS, which have independent prognostic indicators in the multivariate Cox analysis. Each factor is allocated in proportion to its contribution to survival risk (**Figure 9B**). The calibration curve shows that the joint model (nominal chart) shows better accuracy in 1-year and 3-year OS, and the 5-year OS is slightly worse (**Figure 9C**). In summary, these results indicate that the nomogram constructed using the combined model may better predict the short-term survival rate (1 year and 3 years) of patients with STAD than using a single prognostic factor.

### The relationship between tumor risk score and tumor mutation burden

**Figure 10:**
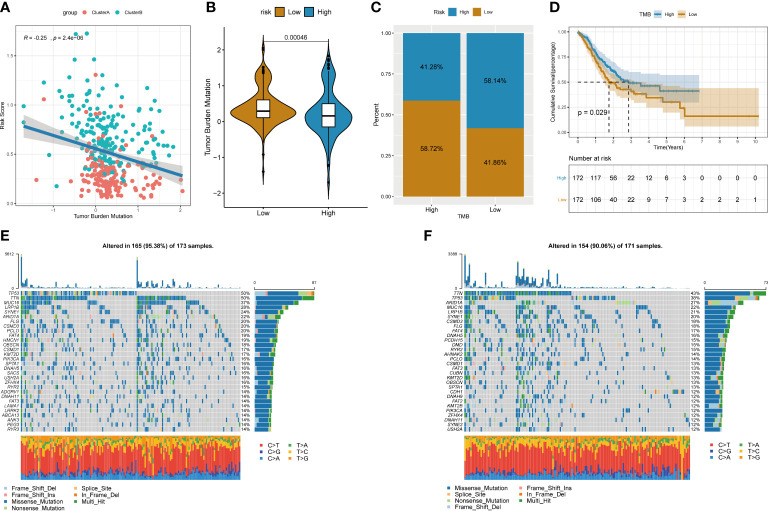
Relationship between tumor risk score and tumor mutational burden. **(A)** Correlation linear regression analysis; **(B)** Violin plot; **(C)**Bar chart of proportional distribution; **(D)**Survival curve; **(E)** Waterfall chart of gene mutation in high risk group; **(F)**Waterfall chart of gene mutation in low risk group.

A large amount of evidence suggests that tumor mutational burden (TMB) may determine the individual response to cancer immunotherapy. Exploring the inner link between TMB and risk score to clarify the genetic characteristics of each angiogenesis subgroup is a meaningful research topic. First, we used the “maftools” package in R to calculate the tumor-free mutation burden (TMB) score. Then, we divided the tumor samples in TCGA_STAD into two groups with high and low TMB scores according to the median TMB. Subsequently, the risk score and TMB were correlated and analysed, and the results showed that the risk score was significantly negatively correlated with TMB (**Figure 10A**). Furthermore, comparing the TMB of patients in the high- and low-risk score groups, the TMB of the subgroup of patients with a higher risk score was significantly lower than that of the subgroup with a lower risk score (**Figures 10B, C**). There were significant differences in survival between the high- and low-risk score groups, and the risk low group had prolonged survival time (**Figure 10D**). In addition, the distribution of somatic variation in STAD driver genes between the low- and high-risk score subgroups was further evaluated, and the top 30 driver genes with the highest change frequency were compared. By analysing the mutation annotation files of the TCGA_STAD cohort, it was found that there were significant differences in mutation profiles between the high- and low-risk score subgroups (**Figures 10E, F**). These results may provide new ideas for studying the mechanism of tumor angiogenesis and gene mutations in immune checkpoints.

### The relationship between the tumor risk score and immune cell infiltration

**Figure 11:**
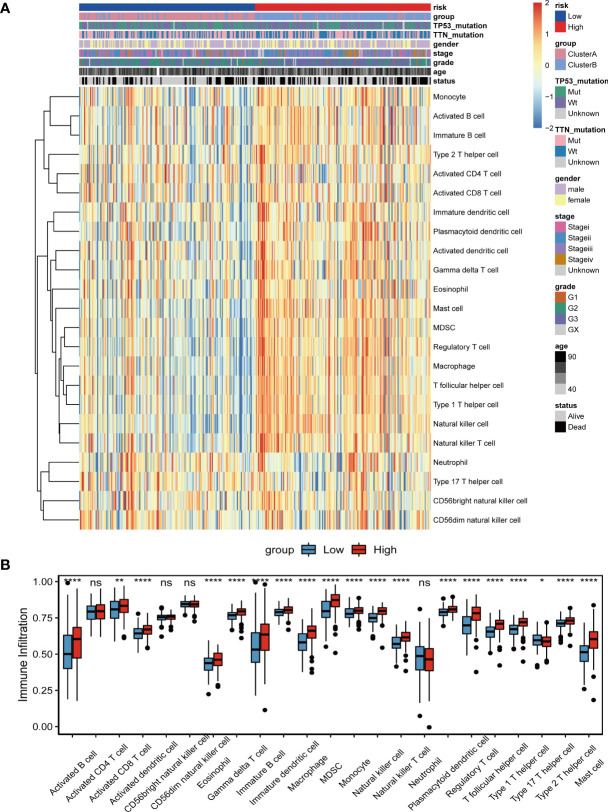
Relationship between tumor risk score and immune cell infiltration. **(A)**The distribution heat map of the proportion of immune cell infiltration between the high and low risk score groups; **(B)** The boxplot of the difference in immune cell infiltration between the high and low risk score groups. Differences between the two groups were analyzed using the independent t-test, values are expressed as the means±sem. ns, P>0.05; *P < 0.05; **P < 0.01; ***P < 0.001; ****P < 0.0001.

To explore the relationship between the risk score of lncRNA construction related to tumor angiogenesis and the tumor immune microenvironment, we used the ssGSEA method to evaluate the infiltration state of 23 immune cells in the TCGA_STAD data set (**Figure 11A**). Then, a hypothesis test was performed on the difference in immune cell infiltration in the high- and low-risk score groups (**Figure 11B**). The comparison found that the degree of infiltration of most immune cells in the high-risk score group was significantly higher than that in the low-risk group, such as activated B cells, activated CD8+ T cells, eosinophils, regulatory T cells, and monocytes. The results indicate that the high risk score may be related to the increase in the degree of immune cell infiltration.

### The predictive ability of the tumor risk score for the benefit of immunotherapy

**Figure 12:**
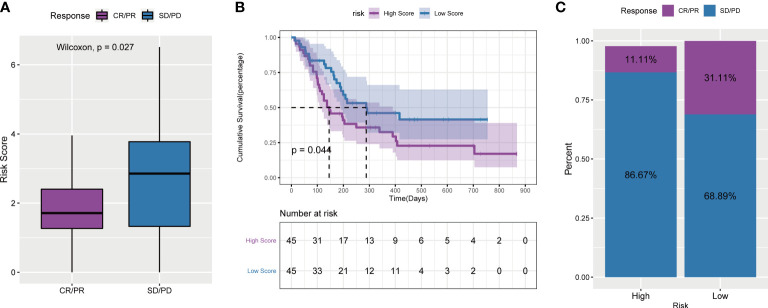
Tumor risk score and prediction of immunotherapy response. **(A)** Comparison of risk scores between responders and non-responders in the ICB immunotherapy cohort; **(B)** Survival curves of high and low risk score groups in the cohort receiving ICB immunotherapy; **(C)** ICB scores in the high and low risk score groups Proportion of patients who responded to immunotherapy.

To explore the predictive ability of the tumor risk score for the benefit of immunotherapy in patients, this study assessed and analysed the GSE176307 data set in the GEO database, in which patients who received ICB immunotherapy were assigned a high or low risk score. It is worth noting that in the ICB immunotherapy response group (CR/PR), the risk score was significantly lower than that in the immunotherapy nonresponse group (SD/PD) (**Figure 12A**). Second, patients in the low-risk score group lived significantly longer than those in the high-risk score group (**Figure 12B**). The objective response rate to ICB immunotherapy in the low-risk score group was higher than that in the high-risk score group (**Figure 12C**).

Above all, the results of these four immunotherapy cohorts confirmed that angiogenesis-related lncRNAs signature had the ability to efficiently predict the efficacy of immunotherapy.

### Expression and functional analysis of six angiogenesis-related lncRNAs in STAD

**Figure 13:**
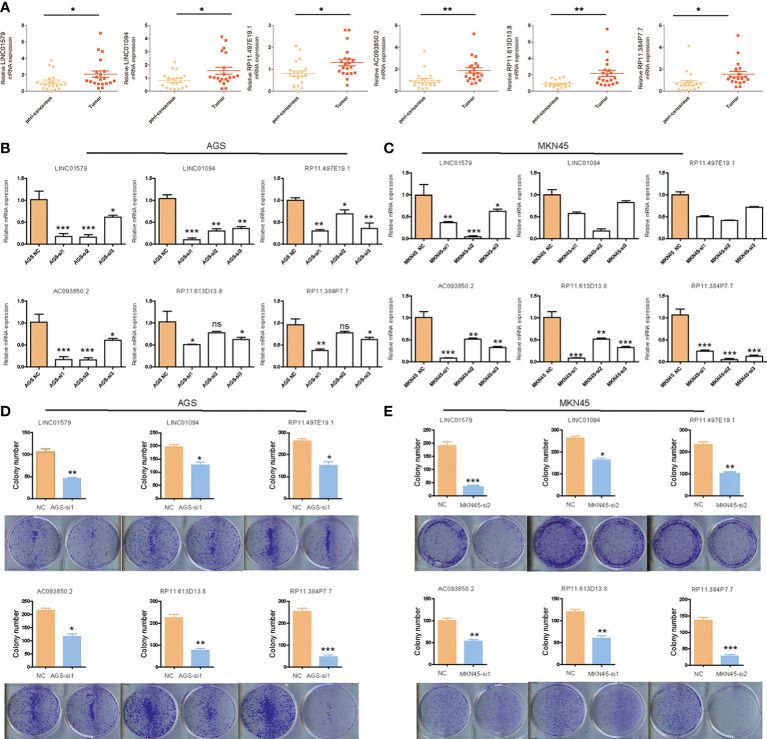
The expression and function analysis of six angiogenesis-related lncRNAs. **(A)** The expression of six angiogenesis-related lncRNAs in STAD by q-PCR; **(B)**The efficiency of knockdown of six angiogenesis-related lncRNAs in AGS cells validated by q-PCR; **(C)**The efficiency of knockdown of six angiogenesis-related lncRNAs in MKN45 cells validated by q-PCR; **(D)**The influence of six angiogenesis-related lncRNAs knockdown on colony-formation ability of AGS cells; **(E)** The influence of six angiogenesis-related lncRNAs knockdown on colony-formation ability of MKN45 cells. Differences between the two groups were analyzed using the independent t-test, values are expressed as the means±sem. ns, P>0.05; *P < 0.05; **P < 0.01; ***P < 0.001; ****P < 0.0001.

To further clarify the expression of six angiogenesis-related lncRNAs, we detected the differences between paired tumor tissues and adjacent nontumor tissues by qRT–PCR. The results showed that the expression of six angiogenesis-related lncRNAs in STAD tissue was higher than that in adjacent tissue (**Figure 13A**). To clarify the functional role of six angiogenesis-related lncRNAs, we knocked down the expression of these lncRNAs in human gastric cancer AGS and MKN45 cells by siRNA, as shown in **Figures 13B, C**. Furthermore, the clone information assay was applied to detect cell proliferation. The results showed that the knockdown of these lncRNAs significantly inhibited the proliferation of ASG and MKN45 cells, as shown in **Figures 13D, E**. Meanwhile, the CCK8 assay was applied to detect cell proliferation. The results showed that the knockdown of these lncRNAs significantly inhibited the proliferation of ASG and MKN45 cells, as shown in **Supplementary Figure S5**.

## Discussion

Angiogenesis is defined as the process of regenerating new blood vessels from an existing capillary network. An “angiogenic switch” is always activated in the tumor, resulting in the constant production of new blood vessels. Tumor-associated neovascularization is a complex physiological event controlled by multiple pro- or antiangiogenic cytokines and multiple signaling pathways, such as vascular endothelial growth factor (VEGF). Despite notable advances in the study of cancer and angiogenesis, research on cancer markers associated with angiogenesis is limited. In this study, we identified an angiogenesis-related gene signature for predicting the prognosis of STAD patients.

Despite many therapeutic advances in recent years, STAD still has a poor prognosis. Current viewpoints highlight that STAD is a heterogeneous malignant illness, and it remains difficult to conduct individualized prognostic evaluations. Therefore, effective molecular biomarkers are critical for improving the prediction and assessment of prognosis in STAD patients. Over the past few decades, many efforts have been made to discover diagnostic and prognostic biomarkers for STAD at the mRNA and miRNA levels (23, 24). However, an increasing number of studies have confirmed that lncRNAs are not only involved in gene regulation but also play an indispensable role in tumor progression (25). High-throughput sequencing analysis has revealed many deregulated lncRNAs in human cancers, and abnormal expression of lncRNAs is related to tumor recurrence and metastasis (10, 26, 27). For example, Ting Yue et al. found that the KCNQ1OT1/miR-378a-3p/RBMS1 axis may be a potential prognostic biomarker and provide insights into the molecular mechanisms of GC pathogenesis (28). LncRNA MAGI2-AS3 is an ideal biomarker and could be a potential therapeutic target for GC (29). LncRNA SLC1A5 may be a novel prognostic marker and a potential target for STAD immunotherapy in the future (30). Although several lncRNA biomarkers were found to help predict the overall survival of patients with STAD, panels of lncRNA biomarkers may be better optimized than single lncRNAs (31, 32).

In the present study, we first analysed the meaningful angiogenesis genes (mRNA) in TCGA_STAD data set. The results showed that low expression of COL5A2, FSTL1, ITGAV, LPL, LUM, NRP1, OLR1, POSTN, SERPINA5, STC1, VCAN and VTN was associated with a better OS prognosis. A total of 289 differentially expressed genes (DES) were identified in the 2 angiogenesis-related genes clusters by consumusclusterplus. A total of 289 DEGs were identified and 116 lncRNAs were screened to have a significant coexpression relationship with angiogenic DEGs. Further, the GSVA results showed that the 2 clusters have significant differences in signal pathways such as DNA replication, mismatch repair, JAK STAT, cytokine receptor interaction, chemokines, TGF BETA, and MAPK pathways. We used Lasso Cox regression analysis to construct the 6 angiogenesis-related lncRNAs signature. The test of internal and external validation of the risk signature results indicated that the risk signature performs well in predicting the risk of STAD patients. The multifactor Cox analysis and Nomogram results showed that our angiogenesis-related lncRNAs signature has good predictive ability for some different clinical factors. Compared to other prognostic signatures, our risk signature focused on angiogenesis-related lncRNAs and constructed a reliable risk model for predicting the effect of immunotherapy in STAD patients (33, 34). For immune, angiogenesis-related lncRNAs signature had the ability to efficiently predict infiltration state of 23 immune cells and immunotherapy. Taken together, these results indicate that this signature is reliable in prognostic prediction and the efficacy of immunotherapy in STAD.

Although tens of thousands of lncRNAs have been identified by transcriptome analysis, their function is still unknown. In this study, we identified 6 novel angiogenesis-related lncRNAs whose functions require further exploration. By consulting the literature, we found that three of the six lncRNAs were associated with tumor progression. Specifically, Chai et al. found that LINC01579 knockdown inhibited cell proliferation and promoted cell apoptosis in glioblastoma by binding with miR-139-5p to regulate EIF4G2 (35). Previous studies have shown that high LINC01094 expression may predict poor prognosis in GC and is correlated with the epithelial-mesenchymal transition pathway and macrophage infiltration (36). In addition, AC093850.2, as a potential prognostic biomarker, promoted migration and proliferation in esophageal squamous cell carcinoma (37). As of now, these are not specifically reported on RP11.497E19.1, RP11.613D13.8 and RP11.384P7.7, and their function remains unknown, thus requiring further studies. To further deepen our understanding of the biological roles of the six lncRNAs, we collected fresh tissue samples of STAD and found that these lncRNAs were highly expressed in cancer tissues compared to adjacent tissues. Meanwhile, we knocked down these lncRNAs by siRNA, which inhibited gastric cancer cell proliferation. These findings revealed important functional roles of the six lncRNAs in tumor progression, and could become potential therapeutic targets in the future.

In this study, we constructed a novel gene signature and conducted extensive analysis to predict the prognosis of STAD patients. The risk signature, based on the expression levels of six angiogenesis-related lncRNA genes, is more cost-effective and clinically feasible than whole-genome sequencing. Meanwhile, the risk signature is useful for predicting the effect of immunotherapy in STAD patients. However, this study also has several limitations. First, this gene signature needs to be further tested in multicenter trials and larger cohorts because we constructed and verified this signature only from a single center. Second, due to technical limitations, we cannot uncover the underlying mechanism research on these lncRNAs. Further experiments need to be conducted to verify our analysis results in the future.

In conclusion, our study identified a novel angiogenesis-related lncRNA signature and constructed a feasible risk model for the prediction of OS in STAD patients, which can be used to evaluate individualized prognosis and the benefit of immunotherapy. These signature key genes related to angiogenesis may serve as new therapeutic targets for STAD patients.

## Data availability statement

The original contributions presented in the study are included in the article/**Supplementary Material**. Further inquiries can be directed to the corresponding authors.

## Ethics statement

The studies involving human participants were reviewed and approved by Renji Hospital Affiliated to Shanghai Jiao Tong University. The patients/participants provided their written informed consent to participate in this study.

## Author contributions

JX, GG and YZ conceived this study. PX, SL and SS performed the experiments. YL, XY, XL and JZ collected samples. PX, SL and SS analyzed the data and drafted the manuscript. All authors contributed to the article and approved the submitted version.

## Supplementary material

Supplementary Figure 1 Flow chart for the research.

Supplementary Figure 2 Effects of TTN gene mutation on angiogenesis-related genes.

Supplementary Figure 3 Effects of TP53 gene mutation on angiogenesis-related genes.

Supplementary Figure 4 Kaplan–Meier analysis on six angiogenesis-related lncRNAs based on TCGA database.

Supplementary Figure 5 The influence of six angiogenesis-related lncRNAs knockdown on CCK8 assay.
