# Supplementary figures and images for: Identification and validation of a novel angiogenesis-related gene signature for predicting prognosis in gastric adenocarcinoma
Source: Front Oncol. 2023 Jan 16;12:965102. doi: 10.3389/fonc.2022.965102 (PMC9885177; doi:10.3389/fonc.2022.965102)

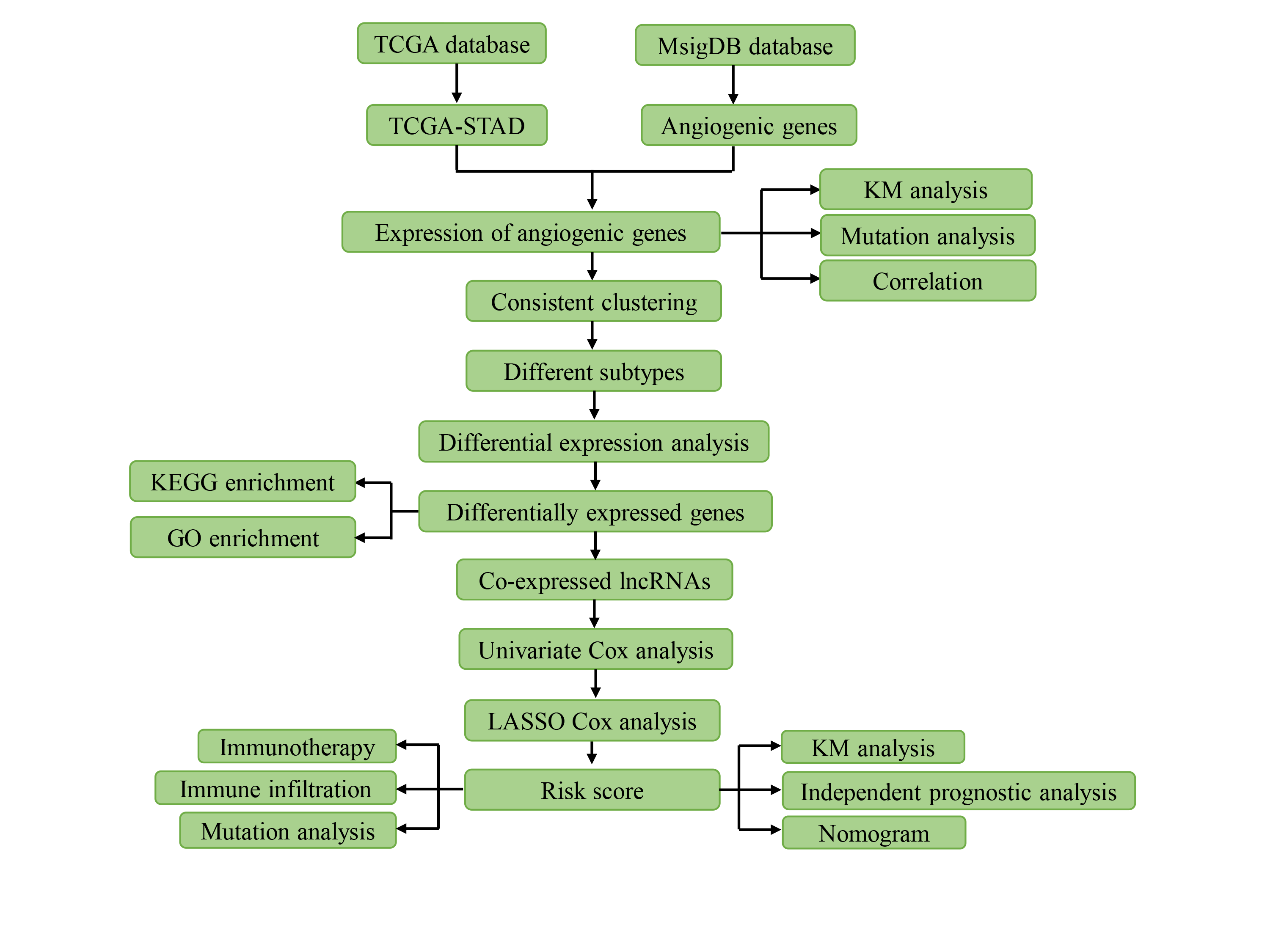

Supplement: Supplementary Figure 1 — Flow chart for the research. [file Image_1.tif]

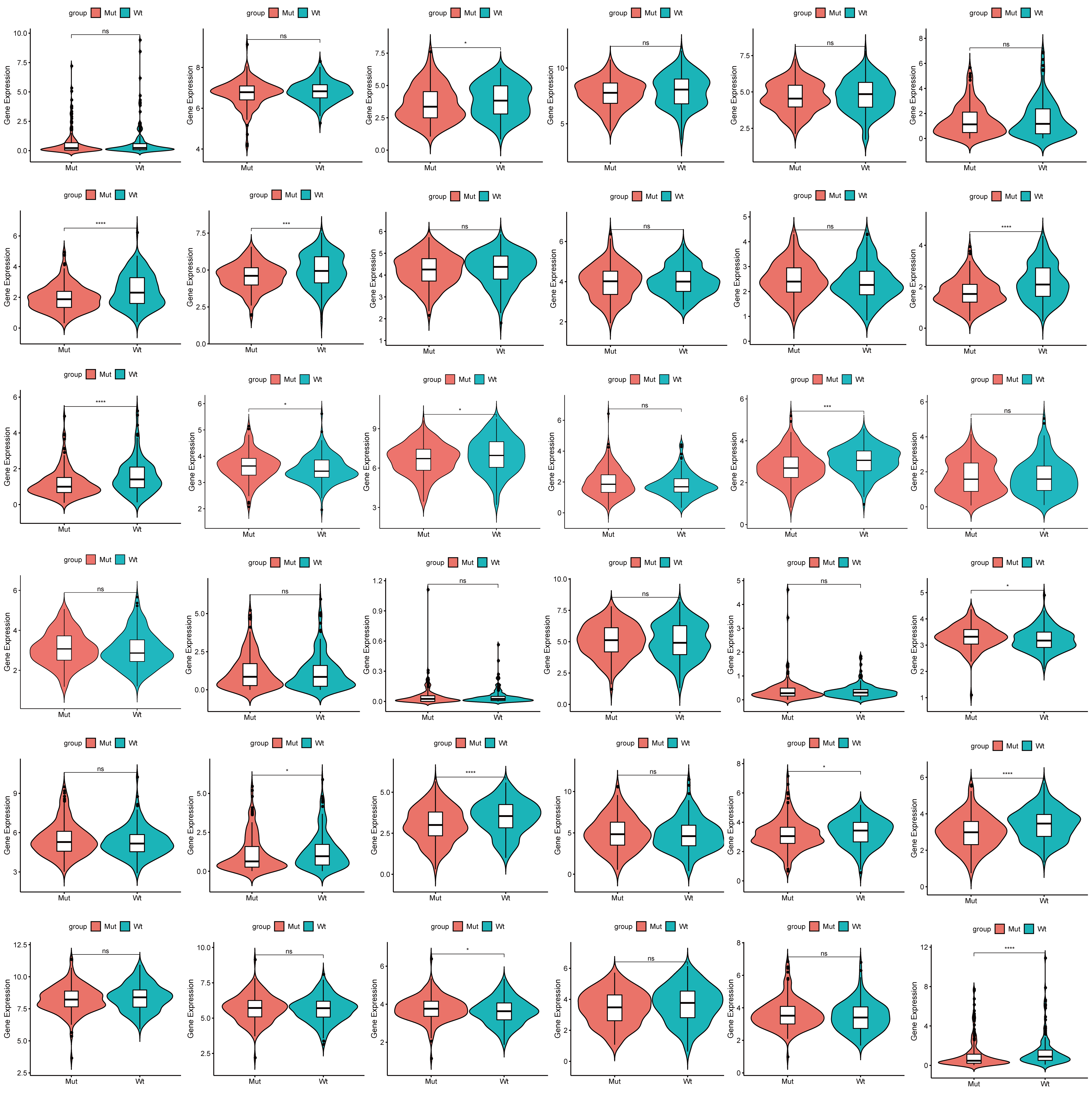

Supplement: Supplementary Figure 2 — Effects of TTN gene mutation on angiogenesis-related genes. [file Image_2.tif]

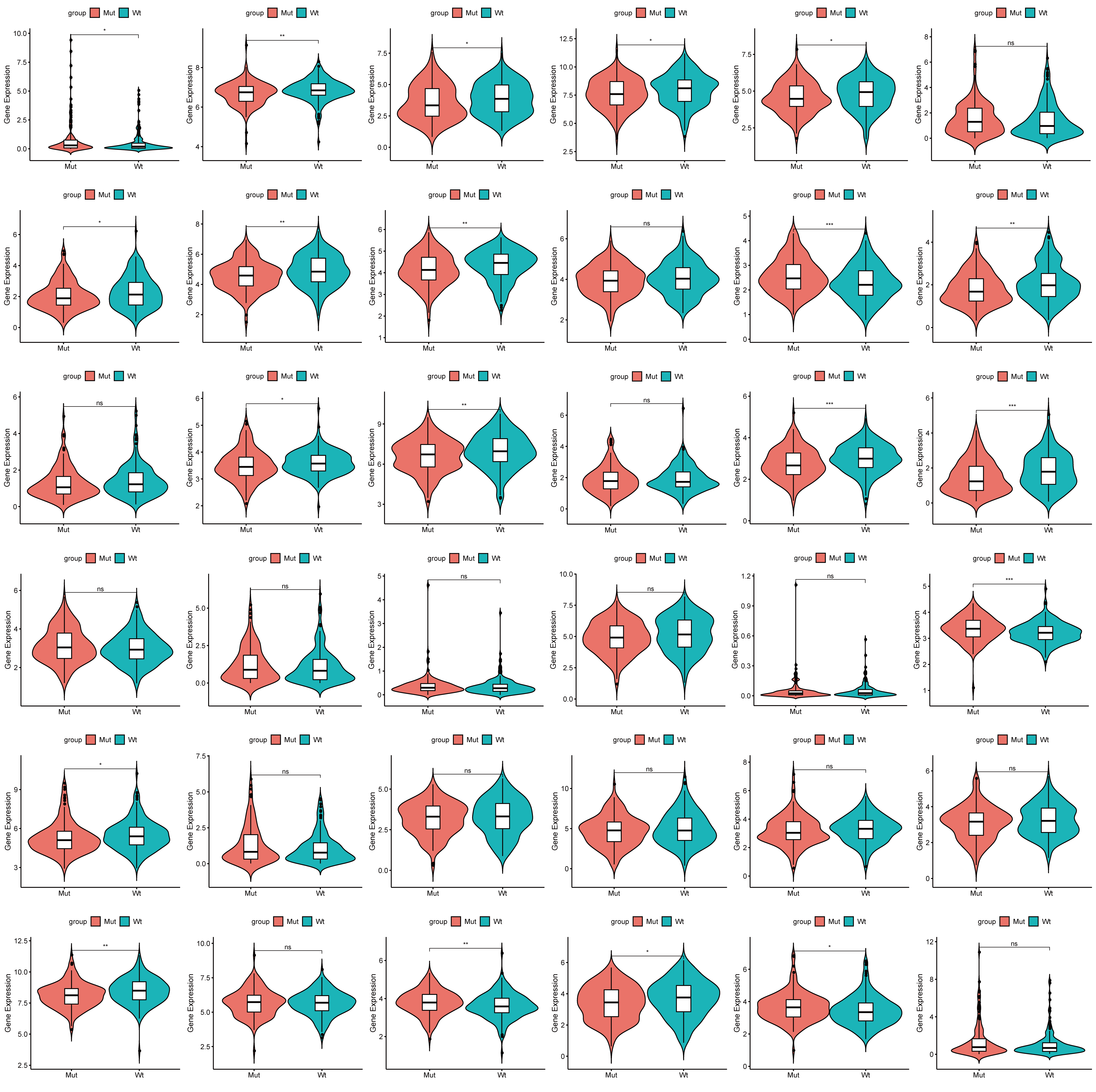

Supplement: Supplementary Figure 3 — Effects of TP53 gene mutation on angiogenesis-related genes. [file Image_3.tif]

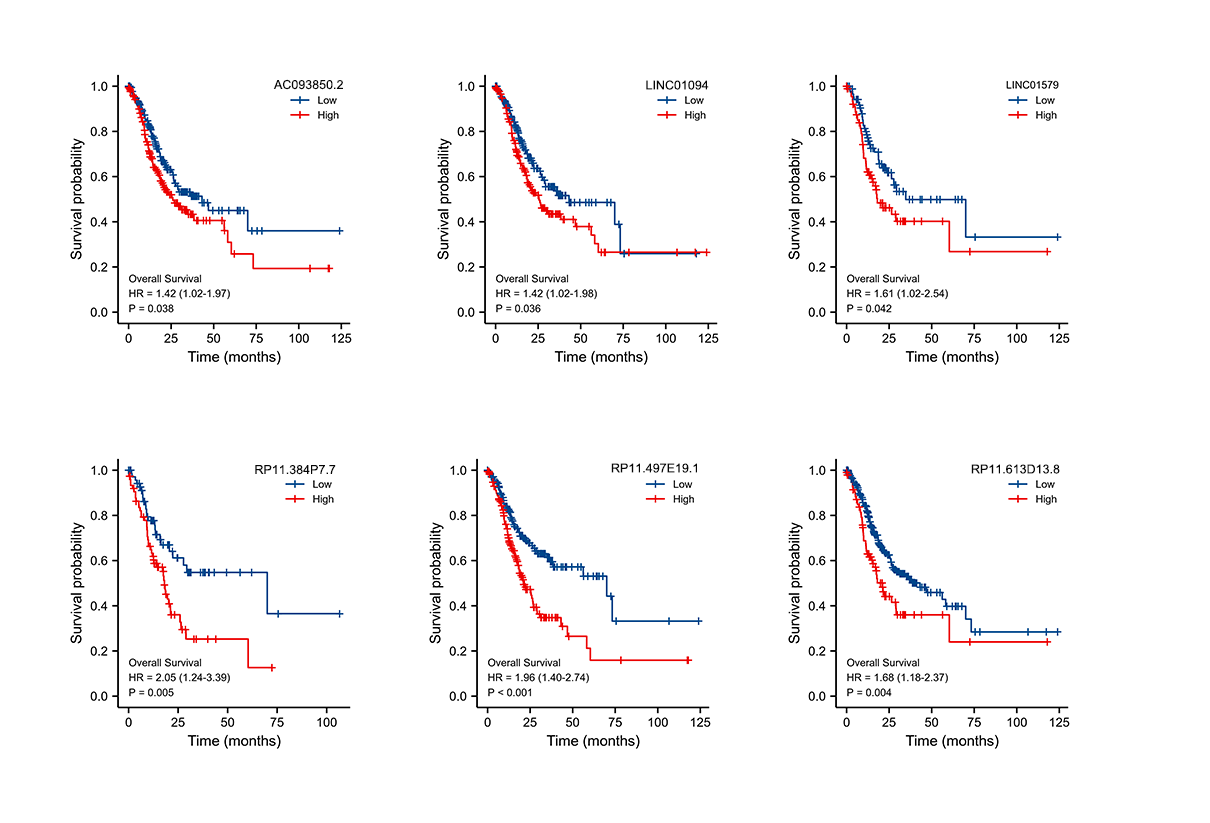

Supplement: Supplementary Figure 4 — Kaplan–Meier analysis on six angiogenesis-related lncRNAs based on TCGA database. [file Image_4.tif]

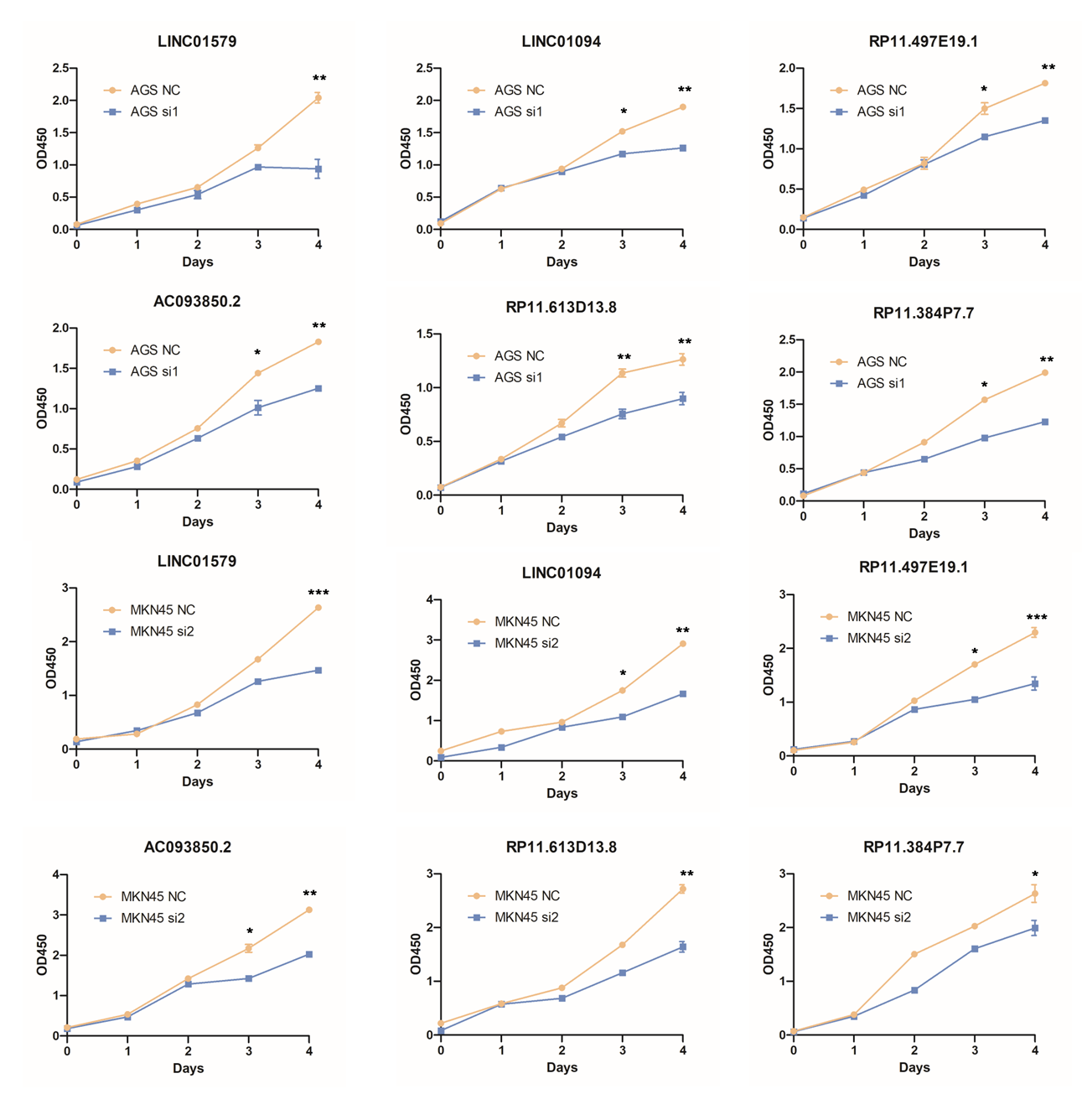

Supplement: Supplementary Figure 5 — The influence of six angiogenesis-related lncRNAs knockdown on CCK8 assay. [file Image_5.tif]
