# Supplementary material for: Identification and validation of a novel angiogenesis-related gene signature for predicting prognosis in gastric adenocarcinoma
Source: Front Oncol. 2023 Jan 16;12:965102. doi: 10.3389/fonc.2022.965102 (PMC9885177; doi:10.3389/fonc.2022.965102)
Supplement: Supplementary file 6 [file Table_1.docx]

Supplementary Table S1: Primers for human specific siRNA

| Genes | sense（5'-3'） | antisense（5'-3'） |
| --- | --- | --- |
| LINC01094 siRNA1 | GUCCCUGAGUUAACAAAUATT | UAUUUGUUAACUCAGGGACTT |
| LINC01094 siRNA2 | GAGGCUGUGUAAUUUAUAATT | UUAUAAAUUACACAGCCUCTT |
| LINC01094 siRNA3 | GUGGGAUGAUACAGAAACUTT | AGUUUCUGUAUCAUCCCACTT |
| LINC01579siRNA1 | CUCCUGAAGAGUAGAAAGATT | UCUUUCUACUCUUCAGGAGTT |
| LINC01579 siRNA2 | CUGGGUAUCUCCUAGAGAUTT | AUCUCUAGGAGAUACCCAGTT |
| LINC01579 siRNA3 | GGCUGGAAUAAAGCAUCGATT | UCGAUGCUUUAUUCCAGCCTT |
| RP11.384P7.7 siRNA1 | GGGCAUACAAAGAGAUAAATT | UUUAUCUCUUUGUAUGCCCTT |
| RP11.384P7.7 siRNA2 | GAGCCUUAGAAUGGAAUAATT | UUAUUCCAUUCUAAGGCUCTT |
| RP11.384P7.7 siRNA3 | CCUCCAGUUACCGGAUUUATT | UAAAUCCGGUAACUGGAGGTT |
| RP11.497E19.1 siRNA1 | GAGCCAGGCAUUUCAAUAATT | UUAUUGAAAUGCCUGGCUCTT |
| RP11.497E19.1 siRNA2 | CCCACAGGUAUAUCUUAAATT | UUUAAGAUAUACCUGUGGGTT |
| RP11.497E19.1 siRNA3 | GCCCAACUCUUAUGUUGAATT | UUCAACAUAAGAGUUGGGCTT |
| RP11.613D13.8 siRNA1 | GCCCAGUCUUUCAGAUUAUTT | AUAAUCUGAAAGACUGGGCTT |
| RP11.613D13.8 siRNA2 | GUGGGUGACUUCAAUUAAATT | UUUAAUUGAAGUCACCCACTT |
| RP11.613D13.8 siRNA3 | CUGCCUGUGAAGGUAUAAATT | UUUAUACCUUCACAGGCAGTT |
| AC093850.2 siRNA1 | GCAGCCAUUUCCCAAAUGUTT | ACAUUUGGGAAAUGGCUGCTT |
| AC093850.2 siRNA2 | GCUCUUUGGAGACUUCAAUTT | AUUGAAGUCUCCAAAGAGCTT |
| AC093850.2 siRNA3 | CCCAAUCAAUUGAUCAUUATT | UAAUGAUCAAUUGAUUGGGTT |
