# Supplementary material for: Identification and validation of a novel angiogenesis-related gene signature for predicting prognosis in gastric adenocarcinoma
Source: Front Oncol. 2023 Jan 16;12:965102. doi: 10.3389/fonc.2022.965102 (PMC9885177; doi:10.3389/fonc.2022.965102)
Supplement: Supplementary file 7 [file Table_2.docx]

Supplementary Table S2: Primers for six angiogenesis-related gene

| Genes | Forward | Reverse |
| --- | --- | --- |
| LINC01094 | CCACGTTGTATGTTTACCCTACC | GCCACTTTCCTCCAGAATTCCA |
| LINC01579 | AGGGACCATTCTGAAGAGCC | ATCTAAGTTCCACGTCACGGC |
| RP11.384P7.7 | GGCAGCAGAGCATAGGGATAG | GCTTCCCTGTGGAACAGACAG |
| RP11.497E19.1 | CACAGGAATCCAGCCTTTTGC | TAGGATGGCTAAAGCAGGGC |
| RP11.613D13.8 | TGCCTGAAAAGCTGGCTAGG | AACACCAGGTTCCAAGGGTG |
| AC093850.2 | AATGGTGGTAGGAGGGAGGA | CAAGGGGAATGAACACGAGG |
